# Modulation of Group I Ribozyme Activity by Cationic Porphyrins

**DOI:** 10.3390/biology4020251

**Published:** 2015-03-24

**Authors:** Shigeyoshi Matsumura, Tatsunobu Ito, Takahiro Tanaka, Hiroyuki Furuta, Yoshiya Ikawa

**Affiliations:** 1 Department of Chemistry, Graduate School of Science and Engineering, University of Toyama, Gofuku 3190, Toyama 930-8555, Japan; E-Mail: smatsumu@sci.u-toyama.ac.jp; 2 Department of Chemistry and Biochemistry, Graduate School of Engineering, Kyushu University, Moto-oka 744, Nishi-ku, Fukuoka 819-0395, Japan; E-Mails: t.itou.048@s.kyushu-u.ac.jp (T.I.); t.tanaka.075@s.kyushu-u.ac.jp (T.T.); hfuruta@cstf.kyushu-u.ac.jp (H.F.); 3 Center for Molecular Systems, Kyushu University, Moto-oka 744, Nishi-ku, Fukuoka 819-0395, Japan

**Keywords:** RNA, ribozyme, porphyrin

## Abstract

The effects of cationic porphyrins on the catalytic activities of four group I ribozymes were investigated. A cationic porphyrin possessing four pyridinium moieties (pPyP) inhibited two group IC3 ribozymes (Syn Rz and Azo Rz) and a group IC1 ribozyme (Tet Rz). In the case of a group IA2 ribozyme (Td Rz), however, pPyP served not only as an inhibitor but also as an activator, and the effects of pPyP were dependent on its concentration. To analyze the structural and electronic factors determining the effects of pPyP on group I ribozymes, three cationic porphyrins (pPyNCP, pPyF4P, and TMPyP) were also examined. As interactions between small organic molecules and nucleic acids are attractive and important issues in biochemistry and biotechnology, this study contributes to the development of porphyrin-based molecules that can modulate functions of structured RNA molecules.

## 1. Introduction

Interactions between nucleic acids and small organic molecules are important issues in the fields of biochemical and pharmaceutical sciences. Small molecule metabolites often regulate the expression of specific genes through direct interaction with their target transcripts bearing aptamer modules [1,2]. The RNA component of the ribosome is one of the major targets of antibiotic small molecules [3,4].

While a variety of small organic molecules have been reported to interact with nucleic acids, aromatic compounds constitute an important class of nucleic acid binding molecules because of their ability to form π-π stacking interactions with nucleobases and base pairs [5,6]. On the other hand, aromatic molecules are hydrophobic and so chemical modifications with positively charged moieties improve their nucleic acid binding ability in aqueous solutions. Positively charged moieties attached to the macrocycles afford not only hydrophilicity but also electrostatic affinity to negatively charged phosphate backbones of nucleic acids. A classical example of this type of molecular structure is ethidium bromide, which has a cationic moiety installed on an aromatic skeleton [7,8].

Among the various classes of aromatic molecules, porphyrins and related tetrapyrrolic macrocycles play indispensable roles in biological systems; they act as cofactors in solar energy conversion (photosynthesis), oxygen transport (myoglobin and hemoglobin), and a number of biological catalysts (enzymes). Naturally occurring porphyrins (including their related macrocycles) usually play their biological roles through interaction with proteins and nucleic acids, suggesting that the porphyrin skeleton is a promising platform for designing nucleic acid binding molecules. Porphyrin derivatives with cationic moieties appended to the *meso*-positions of the macrocycle have been shown to interact with different forms of nucleic acids [9,10,11,12,13,14,15,16,17,18].

Although porphyrins are an attractive class of compounds to modulate and probe functional RNA structures [19,20], only a limited number of studies have been reported to date. Douglas and coworkers reported that several porphyrin derivatives inhibited the processing of precursor tRNAs catalyzed by the RNase P ribozyme from *Escherichia coli* [21]. Celander and Nussbaum employed cationic porphyrins as chemical probes to analyze the higher order structures of functional RNAs involving tRNAs [22]. For further development of porphyrin-based molecules as tools to control RNA structures and functions, it is useful to accumulate information regarding the physical and functional interactions between porphyrins and RNA molecules. In this study, we analyzed group I ribozymes because their interactions with porphyrins have not been explored.

## 2. Experimental Section

### 2.1. Oligonucleotides

DNA oligonucleotides used as PCR primers were purchased from Fasmac (Tokyo, Japan). 5'-Carboxyfluorescein (FAM)-labeled RNA oligonucleotide used as the substrate RNA was purchased from JBIOS (Tsukuba, Japan).

### 2.2. Porphyrin Compounds

Chemical syntheses of pPyP and pPyNCP were reported previously [23]. TMPyP was purchased from Sigma-Aldrich (St. Louis., MO, USA). Synthesis of pPyF4P was carried out using 5,10,15,20-tetrakis{2,3,5,6-tetrafluoro-4-[(methoxymethoxy)methyl]phenyl}-porphyrin (**1**) as a starting material. This porphyrin was obtained as a byproduct (6% yield) in the synthesis of 5,10,15,20,25,30-hexakis{2,3,5,6-tetrafluoro-4-[(methoxymethoxy)methyl]phenyl}-hexaphyrin(1,1,1,1,1,1) [24]. Conversion of the starting porphyrin **1** to pPyF4P was achieved by the procedure developed for synthesis of a water-soluble hexaphyrin. Treatment of porphyrin **1** with H_2_O/trifluoroacetic acid (TFA) mixture promoted the conversion of methoxymethyl groups to hydroxymethyl groups. Four hydroxymethyl groups were further converted to methylene-α-pyridinium groups through treatment with methanesulfonyl chloride in pyridine. Purification of the crude precipitate by reverse phase HPLC with CH_3_CN/H_2_O in the presence of 0.1% TFA gave the target compound pPyF4P as a TFA salt.

### 2.3. Preparation of Ribozymes

Each template DNA for *in vitro* transcription was prepared by PCR with an appropriate plasmid DNA as the template. Plasmid DNAs encoding the four group I intron ribozymes were described previously [25,26,27]. In PCR, a sense primer with a T7 promoter sequence followed by an internal guide sequence (IGS) was used to replace the original P1 region of each ribozyme with IGS. After *in vitro* transcription with T7 RNA polymerase, transcription products were purified by electrophoresis on 6% polyacrylamide gels (29:1 acrylamide:bisacrylamide) containing 7 M urea. The concentrations of RNAs were determined from the absorption at 260 nm (A_260_).

### 2.4. GTP-Dependent Cleavage Reactions Catalyzed by the Group I Ribozymes

Ribozymes dissolved in H_2_O were denatured at 80 °C for 3 min, and then cooled to 37 °C. Tenfold concentrated reaction buffer was added and the resulting solution was incubated for 5 min at 37 °C. Tenfold concentrated porphyrin compound solution was then added and incubations were continued at 37 °C for 5 min in the dark. The reaction was started by adding 5'-FAM-labeled substrate (5'-FAM-GGCCCUCCAAAAA-3') and guanosine triphosphate (GTP; 2 mM final concentration). Final ribozyme and substrate concentrations were 0.1 μM and 1.0 μM, respectively. The final reaction buffer contained 30 mM Tris-HCl (pH 7.5) and 50 mM MgCl_2_ (or 5 mM MgCl_2_ for Tet Rz). In the activity assay of the group I ribozymes, monovalent cations (Na^+^, K^+^, NH_4_^+^) often cause positive and negative effects on the activity of each ribozyme in an unpredictable manner although they are not essential for catalysis. Therefore, the monovalent cations were omitted from the reaction buffer to simplify and unify the buffer composition used in this study. The reactions were carried out at 37 °C in the dark, and aliquots were taken at specified times and treated with an equal volume of stop solution consisting of 80% formamide and 100 mM EDTA. Products and substrates were separated on 15% polyacrylamide gels (29:1 acrylamide:bisacrylamide) containing 7 M urea. The intensities of the bands were quantified by FluoroImager Pharos FX (BioRad, Hercules, CA, USA). The data were fitted to the following equation: Fraction reacted = F_a_(1 − e^−kt^), where t is time. The initial rates of the cleavage reactions of the substrate were calculated as F_a_k. All experiments were repeated at least twice. The mean values are shown in the figures, and error bars indicate the minimal and maximal values.

## 3. Results

**Figure 1:**
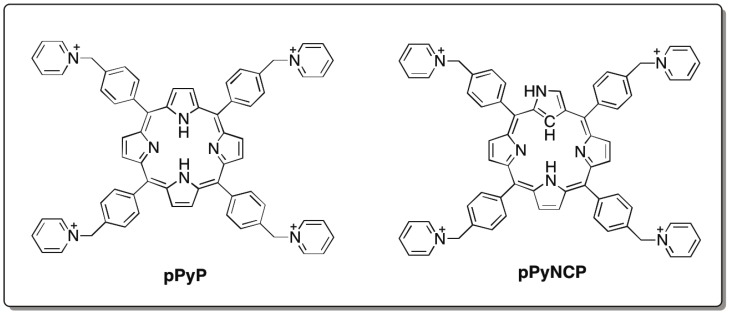
Chemical structures of the cationic porphyrin (pPyP) and the cationic *N*-confused porphyrin (pPyNCP).

To investigate interactions between porphyrins and group I ribozymes, we employed *meso*-tetraarylporphyrins (Figure 1) as a core platform structure because they have been used frequently in designing porphyrin-based functional molecules [9,10,11,12,13,14,15,16,17,18,19,20,21,22,23,24]. In this study, we primarily used pPyP and pPyNCP (Figure 1) [23,28]. pPyP and pPyNCP have been shown to interact with various forms of DNA molecules, including single- and double-stranded DNAs [23,28]. The functional effects of cationic porphyrins on group I ribozymes were evaluated by monitoring the ribozyme activities in the presence of different amounts of the porphyrin compounds.

### 3.1. Effects of Cationic Porphyrins on Group IC3 Ribozymes

**Figure 2:**
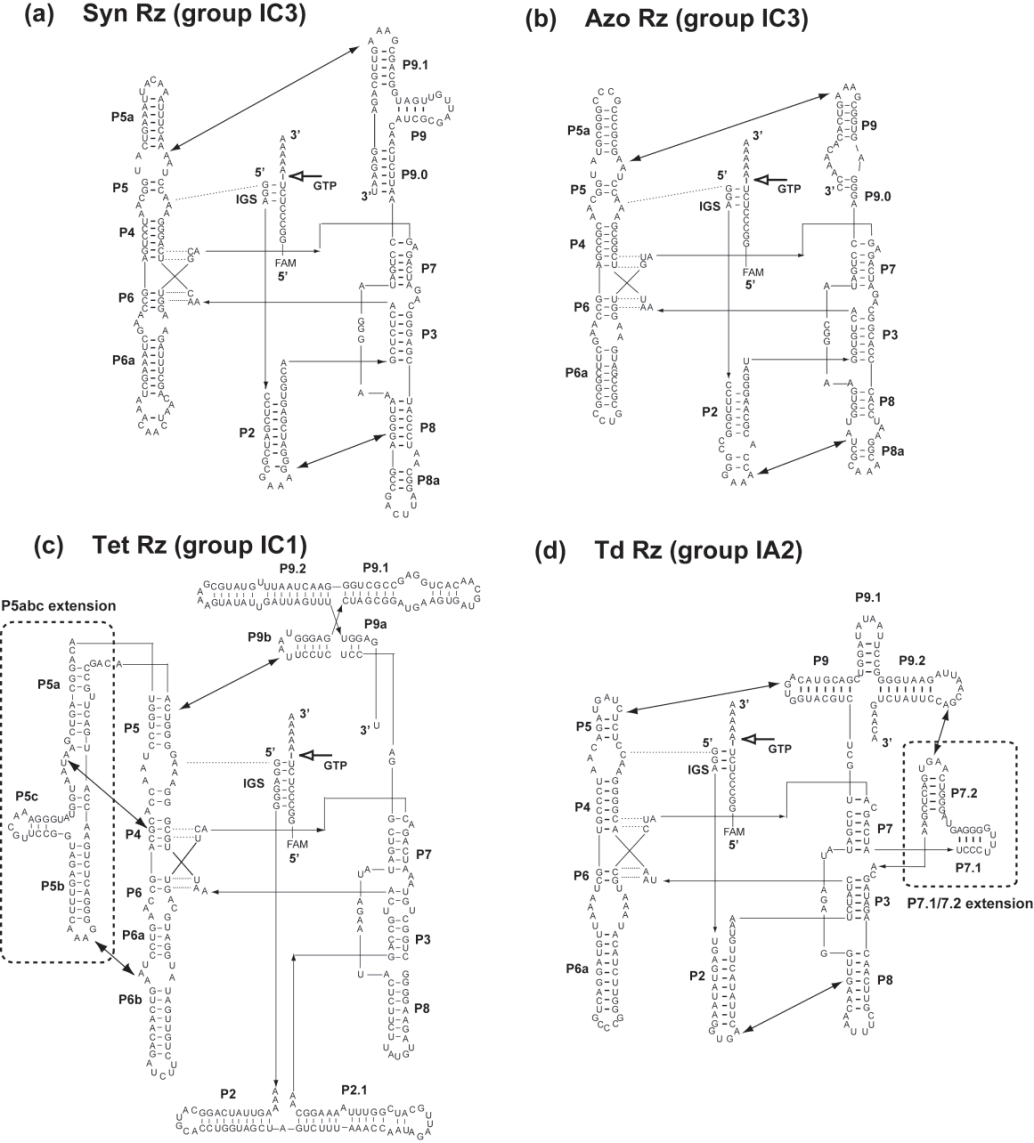
Secondary structures of four group I ribozymes employed in this study. They are Syn group IC3 Rz (**a**), Azo group IC3 Rz (**b**), Tet group IC1 Rz (**c**), and Td group IA2 Rz (**d**). Each ribozyme cleaves a common substrate RNA, which is recognized by the IGS element in each ribozyme. Solid lines with two closed arrows indicate tertiary interactions. Broken lines indicate base triplet interactions that contribute to recognition of the cleavage site by the catalytic core and support the assembly of two core domains. White arrows indicate the GTP-dependent cleavage site in the substrate RNA. Regions enclosed with broken lines indicate the P5abc extension in Tet Rz (**c**) and the P7.1/7.2 extension in Td Rz (**d**).

Group I ribozymes share a conserved core structure formed through assembly of two helical elements, *i.e.*, P4-P5-P6 and P7-P3-P8 [29,30]. Group I ribozymes also have peripheral elements that are structurally diverse among intron subgroups [29,30]. We first employed two closely related group IC3 ribozymes from bacterial tRNA precursors from *Synechococcus* PCC6301 (Syn Rz) and *Azoarcus* sp. HB72 (Azo Rz) (Figure 2a,b) [27]. They share highly analogous secondary structures in their core and peripheral elements. On the other hand, they also have a marked difference in their nucleotide composition. The nucleotide sequence of Azo Rz is highly GC-rich, and so the content of G-C base pairs in its secondary structure is higher than that of Syn Rz (Figure 2b).

In cellular contexts, group I ribozymes exist as introns in primary transcripts and perform self-splicing (excision of themselves from primary transcripts) [31]. In the self-splicing reaction of group I ribozymes, the internal guide sequence (IGS) serves as a major determinant of the 5' splice site. We modified the self-splicing ribozymes to catalyze site-specific cleavage of a short substrate RNA (Figure 2). This modification is a standard method for evaluation of the catalytic ability of group I ribozymes. In this format, group I ribozyme can behave as a true catalyst, in which IGS recognizes the substrate RNA by sequence complementarity leading to substrate cleavage [32,33,34,35].

**Figure 3:**
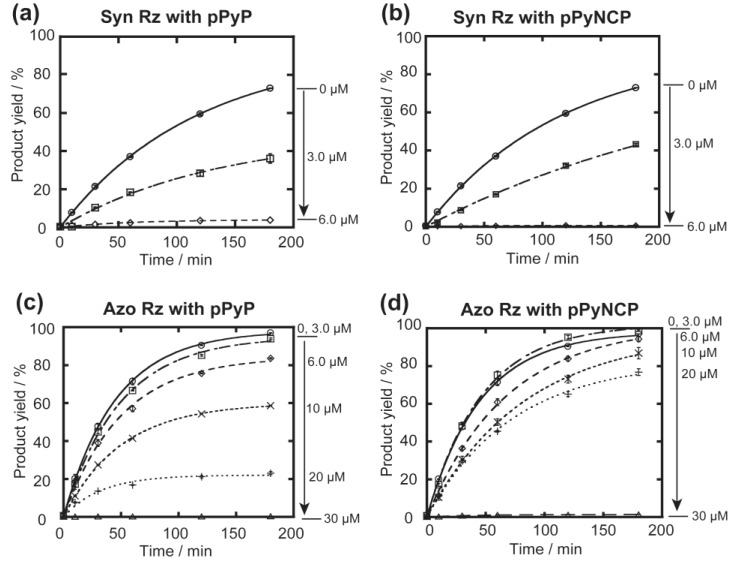
Effects of cationic porphyrins on the GTP-dependent cleavage reactions catalyzed by the two group IC3 ribozymes. Reactions were carried out with 0.1 µM ribozyme, 1.0 µM 5'-FAM-labeled substrate RNA, 50 mM Mg^2+^, and 30 mM Tris-Cl (pH 7.5) at 37 °C. (**a**,**b**) Time courses of the cleavage reactions catalyzed by the Syn ribozyme in the presence of (**a**) pPyP and (**b**) pPyNCP; (**c**,**d**) Time courses of the cleavage reactions catalyzed by the Azo ribozyme in the presence of (**c**) pPyP and (**d**) pPyNCP.

In the presence of 2 mM guanosine triphosphate (GTP) serving as a nucleophile in the cleavage reaction (Figure 2) and 50 mM Mg^2+^ ions contributing to RNA folding and catalysis, a catalytic amount (0.1 μM) of Syn Rz and Azo Rz showed multiple cleavage of the substrate RNA (1.0 μM) (Figure 3). Addition of pPyP and pPyNCP inhibited the cleavage reaction. At 6.0 μM, pPyP completely inhibited the cleavage reaction by Syn Rz (Figure 3a). In the reaction with Syn Rz, pPyNCP also acted as an inhibitor but was slightly less effective than pPyP although RNA cleavage by Syn Rz was markedly inhibited by 6.0 μM pPyNCP (Figure 3b). These results indicated that the two compounds bind to Syn Rz and inhibit its catalytic ability. In the presence of 3.0 μM pPyP, the observed rate constant of substrate cleavage by Syn Rz (0.36 × 10^−2^ min^−1^) was 2.2-fold slower than that without the macrocycle (0.79 × 10^−2^ min^−1^) (Figure 3a).

The same experiment was then carried out with Azo Rz, the IGS of which was also engineered to cleave the same substrate (Figure 3c,d). Under reaction conditions identical to those for Syn Rz, cleavage of the substrate by Azo Rz (2.2 × 10^−2^ min^−1^) was 2.7-fold more rapid than that by Syn Rz (0.79 × 10^−2^ min^−1^). Consistent with the Syn Rz-catalyzed cleavage reaction, pPyP and pPyNCP inhibited the catalytic ability of Azo Rz (Figure 3c,d). However, Azo Rz was much more tolerant of inhibition by porphyrins because 6.0 μM pPyP inhibited Az Rz only modestly (Figure 3c). Catalytic activity of Azo Rz was still observed even with 20 μM porphyrins. Azo Rz also clarified the difference between pPyP and pPyNCP in inhibition ability. pPyP with regular porphyrin core served as a more effective inhibitor of Az Rz than pPyNCP with N-confused porphyrin core (Figure 3c,d). This difference suggests a contribution of hydrophobic interaction in the association between Azo Rz and porphyrin macrocycles because N-confused porphyrin is more hydrophilic than regular porphyrin because of its outward pointing N atom in the confused pyrrole unit.

### 3.2. Effects of the Cationic Porphyrin on the Group IC1 Ribozyme

To determine whether the inhibitory effect of pPyP on group I ribozymes is specific to the two analogous group IC3 ribozymes, we next examined a group I ribozyme from *Tetrahymena thermophila* ribosomal RNA precursor (Tet Rz, Figure 2c). Tet Rz belongs to the group IC1 ribozymes with larger and more complex structures than group IC3 ribozymes. Group IC1 ribozymes share a large peripheral element P5abc that extends from the core P5 element (Figure 2c). Tet Rz is highly active due to its stable three-dimensional structure supported by multiple tertiary interactions between the core elements and P5abc extension.

**Figure 4:**
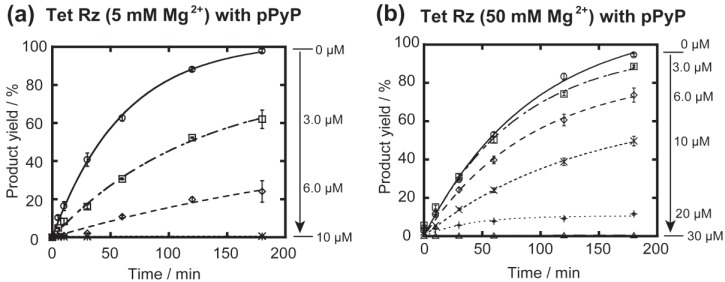
Effects of the cationic porphyrin pPyP on the GTP-dependent cleavage reactions catalyzed by the Tet ribozyme. Reactions were carried out with 0.1 µM ribozyme, 1.0 µM 5'-FAM-labeled substrate RNA, 5 or 50 mM Mg^2+^, and 30 mM Tris-Cl (pH 7.5) at 37 °C. (**a**) Time course of the cleavage reaction catalyzed by the Tet ribozyme in the presence of pPyP and 5 mM Mg^2+^; (**b**) Time course of the cleavage reaction catalyzed by the Tet ribozyme in the presence of pPyP and 50 mM Mg^2+^.

Tet Rz is fully active under low concentration (5 mM) of Mg^2+^ ions due to its structural stability and also its IGS forming longer base pairs than other ribozymes employed in this study (Figure 2). In the presence of 5 mM Mg^2+^ and 2 mM GTP, catalytic cleavage of the substrate (1.0 μM) by Tet Rz (0.1 μM) proceeded smoothly (Figure 4a). The dose-dependent inhibitory effect of pPyP suggests that the tolerance of Tet Rz to pPyP was higher than Syn Rz but lower than Azo Rz.

To determine the effects of Mg^2+^ ions that serve as general stabilizers of RNA tertiary structures, the inhibitory effect of pPyP on Tet Rz folded in the presence of 50 mM Mg^2+^ ions was examined (Figure 4b). In the absence of pPyP, the initial rate of the reaction by Tet Rz with 50 mM Mg^2+^ ions (1.2 × 10^−2^ min^−1^) was slightly lower than that with 5 mM Mg^2+^ ions (1.7 × 10^−2^ min^−1^) probably because a higher concentration of Mg^2+^ ions slows ribozyme turnover by stabilizing the ribozyme-product complex and/or reduces the population of active ribozyme by stabilizing misfolded structures. In the presence of 50 mM Mg^2+^ ions, Tet Rz showed higher tolerance to pPyP than that with 5 mM Mg^2+^ ions because Tet Rz was partially active even in the presence of 10 μM pPyP. The catalytic activity with 10 μM pPyP also suggested that Tet Rz was less tolerant to pPyP than Azo Rz.3.3. Effects of Cationic Porphyrins on the Group IA2 Ribozyme

**Figure 5:**
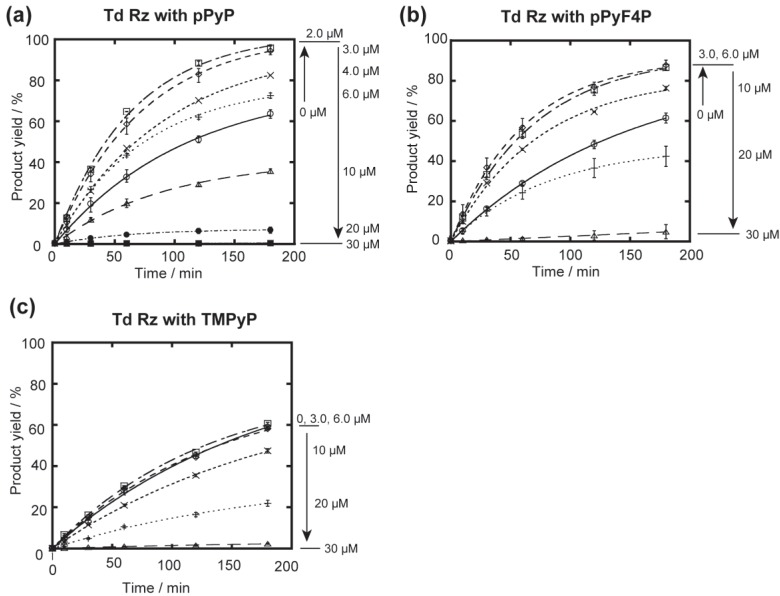
Effects of cationic porphyrins on the GTP-dependent cleavage reactions catalyzed by the Td ribozyme. Reactions were carried out with 0.1 µM ribozyme, 1.0 µM 5'-FAM-labeled substrate RNA, 50 mM Mg^2+^, and 30 mM Tris-Cl (pH 7.5) at 37 °C. (**a**) Time courses of the cleavage reactions catalyzed by the Td ribozyme in the presence of pPyP; (**b**) Time courses of the cleavage reactions catalyzed by the Td ribozyme in the presence of pPyF4P; (**c**) Time courses of the cleavage reactions catalyzed by the Td ribozyme in the presence of TMPyP.

As an additional example of group I ribozymes bearing large peripheral elements, we chose the Td group IA2 ribozyme from bacteriophage T4 (Td Rz, Figure 2d). The secondary structure of Td Rz is similar to those of Syn Rz and Azo Rz except for a large extension (P7.1/7.2) inserted between core P7 and P3 elements. In the three-dimensional structure, the P7.1/7.2 extension associates with the P7-P3-P8 element in the core region, whereas the P5abc extension of Tet Rz docks with the P4-P5-P6 element. In the presence of 50 mM Mg^2+^ ions and 2 mM GTP, Td Rz (0.1 μM) catalytically cleaved the substrate RNA (1.0 μM) (Figure 5a) with efficiency (0.71 × 10^−2^ min^−1^) comparable to that of Syn Rz (0.1 μM, 0.79 × 10^−2^ min^−1^, Figure 3a).

In contrast to the three ribozymes tested previously, Td Rz characteristically responded to pPyP. While pPyP only showed inhibitory effects on Syn, Azo, and Tet ribozymes, 2.0–6.0 μM pPyP enhanced the cleavage reaction by Td Rz (Figure 5a). This positive effect was particularly remarkable with 2.0–3.0 μM compounds, with which the rate constant for Td Rz catalyzed cleavage (1.4–1.6 × 10^−2^ min^−1^) was twice that without pPyP (0.71 × 10^−2^ min^−1^). Enhancement of Td Rz activity was similarly observed in the cleavage reaction in the presence of 2.0–6.0 μM pPyNCP [36].

**Figure 6:**
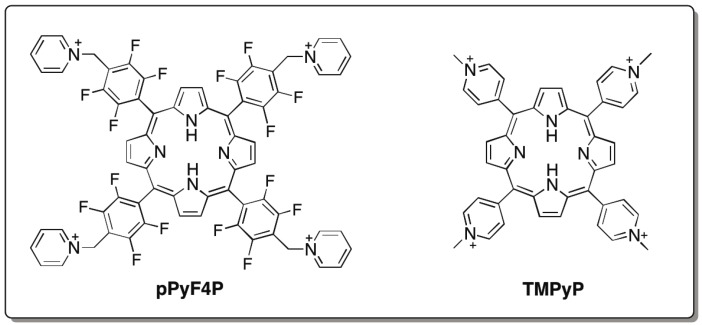
Chemical structures of two cationic porphyrins (pPyF4P and TMPyP).

To investigate the elements in pPyP (and pPyNCP) contributing to the improvement of Td Rz catalytic activity, we examined two additional porphyrin compounds, pPyF4P and TMPyP (Figure 6). pPyF4P was employed to modify the steric and electronic properties of pPyP without altering the relative positions of four cation charges. For this purpose, four fluorine atoms were introduced to each *meso*-aryl moiety of pPyP (Figure 1). In the resulting molecule (pPyF4P), the porphyrin core became less electron-rich and more hindered because *meso*-aryl groups were bulkier and more electron-withdrawing than those of the parent pPyP. In the presence of 3.0–10 μM pPyF4P, the activity of Td Rz was higher than that without the porphyrin (Figure 5b), indicating that the activating effect of pPyF4P on the Td Rz reaction is similar to that of pPyP. On the other hand, the inhibitory effects of pPyF4P on Td Rz were weaker than those of pPyP. At 10 μM, pPyF4P still activated Td Rz, whereas 10 μM pPyP inhibited the reaction (Figure 5b). The initial reaction rate with 20 μM pPyF4P (0.58 × 10^−2^ min^−1^) was comparable to that without porphyrin (0.71 × 10^−2^ min^−1^), whereas 20 μM pPyP almost completely inhibited the reaction.

TMPyP sharing four pyridinium groups to pPyP was employed to alter the relative positions of four cationic charges (Figure 1). In the chemical structure of TMPyP, pyridinium cations are closer to the tetrapyrrole skeleton than those in pPyP (Figure 1). In the presence of 3.0 μM TMPyP, the activity of Td Rz was nearly the same as that without the porphyrin compound (Figure 5c). In the presence of a higher concentration of TMPyP (>3.0 μM), the activity of Td Rz was inhibited. The inhibitory effect of TMPyP was weaker than that of pPyP (Figure 5c). These results suggest that the chemical structure surrounding the periphery of the porphyrin macrocycle has a significant impact on the functional interaction of the cationic porphyrin and Td Rz. The positions of pyridinium cations are important for the activating effect by cationic porphyrins, whereas their core structures and aryl groups primarily govern their inhibitory effect on Td Rz.

## 4. Discussion

This study demonstrated that cationic porphyrins serve not only as inhibitors but also as activators for RNA catalysts. Although the molecular basis underlying the bidirectional effects of pPyP on Td Rz is unknown, the P7.1/7.2 extension may be a possible target of pPyP. This speculation should be validated experimentally by comparing the effects of pPyP on wild-type Td Rz and its mutant derivatives with deletion of the P7.1/7.2 element. With regard to the molecular mechanism underlying the positive effect of pPyP at low concentrations, pPyP may act like polyamines that can enhance the catalytic ability of weakly active group I ribozymes. Two group IC3 ribozymes and Tet Rz were inhibited by pPyP, suggesting that the inhibitory effect of pPyP is independent of the coverage of the P4-P5-P6 helical core domain by P5abc extension in Tet Rz. These results suggest that the possible target region of pPyP and other porphyrins is the P7-P3 domain rather than the P4-P5-P6 domain in the conserved core elements of group I introns. Chemical probing experiments are required to clarify the molecular basis of porphyrin-ribozyme interactions, including identification of porphyrin-binding sites within each ribozyme.

In the present and previous studies, naturally occurring ribozymes were tested as nucleic acid targets of common cationic porphyrins (such as TMPyP and pPyP). On the other hand, a customized ligand for G4-DNAs has been designed based on an expanded porphyrin (pentapyrrolic sapphyrin) [37]. This example encourages us to design specific ligands for given ribozymes (and other structured RNAs) through rational or combinatorial approaches. Both approaches would be possible through modular assembly of porphyrin (including its isomers and analogs) cores and peripheral moieties.

The importance of physical and functional interactions between porphyrin compounds and RNA catalysts has been recognized from the viewpoint of the early evolution of living systems. As porphyrin pigments play indispensable roles in modern biosystems, including photosynthesis, its emergence and biosynthesis in the early stages of life are important issues. In the protein-based heme (Fe(II) complex of protoporphyrin XI) biosynthesis, the last step (Fe-insertion to protoporphyrin XI) has been mimicked by artificial RNA catalysts [38]. Protein-assisted peroxidase activity of heme was also mimicked with the assistance of RNA aptamers [39,40,41]. In an early step of a modern porphyrin biosynthetic pathway, glutamyl-tRNA was employed as a precursor of the pyrrole unit [42]. These observations suggest that porphyrin biosynthesis emerged in the RNA world, in which RNA catalysts may have synthesized tetrapyrrole compounds [43]. Although marked acceleration of chemical transformation by enzymatic catalysts is achieved through highly sophisticated catalytic sites, primitive forms of catalysts modestly promote the reaction through simple physical interactions with substrates. Porphyrin-dependent modulation of RNA-catalyzed RNA-processing reactions (involving group I ribozyme reactions and RNase P ribozyme-catalyzed tRNA processing) suggests that complex tertiary RNA structures are suitable to provide binding pockets for porphyrins and their precursors. A systematic survey of porphyrin-RNA interactions would provide deeper insight into the evolutionary origin of porphyrin biosynthesis. To further analyze modern and evolutionary aspects of porphyrin-RNA interactions, we are currently designing and analyzing cationic porphyrins and their related macrocycles.

## 5. Conclusions

In this study, we investigated the functional effects of pPyP and related cationic porphyrins (pPyNCP, pPyF4P, and TMPyP) on the catalytic activities of group I ribozymes. To our knowledge, this is the first report on the interaction between porphyrins and group I ribozymes. Cationic porphyrins target various forms of DNA molecules, and often lead to inhibition of DNA-protein interactions. For example, TMPyP interacts with telomeric G-quadruplex structures and inhibited elongation of telomeric repeats catalyzed by telomerase [11]. TMPyP also inhibits the site-specific cleavage of tRNA precursors catalyzed by the RNase P ribozyme [21]. These results taken together with our observations that pPyP and pPyNCP inhibit the group I ribozymes suggest that porphyrins and their related macrocycles are promising platforms to develop novel inhibitors of functional RNAs.
